# Association of Serum Lipocalin-2 Concentrations with Psoriasis and Psoriatic Arthritis: An Updated Meta-Analysis

**DOI:** 10.1155/2019/7361826

**Published:** 2019-08-05

**Authors:** Dingjian Wang, Lanlan Fang, Guixia Pan

**Affiliations:** Department of Epidemiology and Biostatistics, School of Public Health, Anhui Medical University, 81 Meishan Road, Hefei, Anhui 230032, China

## Abstract

**Objectives:**

The purpose of this study was to explore the association of serum lipocalin-2 concentrations with psoriasis and psoriatic arthritis (PsA).

**Methods:**

A systematic search of studies on the association of serum lipocalin-2 concentrations with psoriasis/PsA was conducted in PubMed, Web of Science, Elsevier ScienceDirect, and Cochrane Library. Eventually, 8 eligible studies were included. The strength of association between serum lipocalin-2 concentrations and psoriasis/PsA was assessed by pooled standard mean difference (SMD) with its 95% confidence intervals (CIs).

**Results:**

A total of 8 case-control studies, consisting of 349 psoriasis/PsA patients and 258 controls, were included in the meta-analysis. This meta-analysis showed significant association between serum lipocalin-2 concentrations and psoriasis/PsA in overall population (SMD: 0.757, 95%CI = 0.588‐0.926, and *P*_H_ = 0.114; *P*_H_ is the *P* value for the heterogeneity test). Similar results were found in subgroup analysis by ethnicity.

**Conclusions:**

Serum lipocalin-2 concentrations are higher in psoriasis/PsA patients than controls. However, more large-scale studies are warranted to explore the association between serum lipocalin-2 and the pathogenetic mechanisms of psoriasis/PsA.

## 1. Introduction

Psoriasis is a common, chronic, immune-mediated, inflammatory skin disease characterized by the formation of well-demarcated, erythematous plaques with silvery scales [1–3]. The etiology of psoriasis is very complex, which is affected by both genetic and environmental factors. The incidence and prevalence of psoriasis are also significantly different due to region and ethnicity [4, 5]. It has been related to obesity, metabolic syndrome, and cardiovascular disease [6]. In addition, psoriasis has also been recognized as an independent risk factor for myocardial infarction, which may cause serious negative impact on the quality of life [7]. Psoriatic arthritis (PsA) is a chronic, inflammatory, and debilitating arthritis associated with psoriasis [8]. The etiology of psoriasis and PsA is unknown; some studies have shown that adipokines and cytokines may play an important role in its pathogenetic mechanisms [9, 10].

Lipocalin-2 (LCN2), also known as neutrophil gelatinase-associated lipocalin (NGAL), is a member of the lipocalin family of proteins [11, 12]. LCN2 is expressed in various tissues or cells, including liver, lung, kidney, adipocytes, macrophages, and epithelial cells [13]. It has been identified as a proinflammatory mediator to enhance the production of important cytokines, such as IL-6, IL-8, and CXCL10, resulting in aggravating the diseases [14]. Also, LCN2 is an antimicrobial protein; it has been reported that LCN2 may play a key role in the innate immune response to bacterial infection [15]. In addition to initiating innate immune responses, LCN2 modulates cellular immunity and inflammation. Hence, the functions of LCN2 extend beyond those of antimicrobials, and it has also been implicated in the progression of various diseases including infectious disease, cardiac disease, renal injury, tumor metastasis, and chronic inflammatory pain, among others [16–18]. Moreover, some studies have reported that serum LCN2 levels are increased in psoriasis patients than controls [19, 20]. LCN2 is suggested to be involved in the pathogenesis of psoriasis/PsA by modulating neutrophil function and enhancing T-helper 17 response [21, 22].

Compared with the study conducted by Bai et al. [5], first, our meta-analysis explores the association of serum lipocalin-2 concentrations with psoriasis and psoriatic arthritis, not just about psoriasis. Second, our meta-analysis emphasizes the mechanism between lipocalin-2 and psoriasis/psoriatic arthritis. Third, the number of studies in our meta-analysis is more than the study conducted by Bai et al. Considering that the results of serum lipocalin-2 concentrations with psoriasis/PsA are inconsistent [23], this discrepancy might be due to studies with small sample size, inadequate statistical power, ethnic differences, and publication bias. Therefore, it is necessary to conduct a meta-analysis to explore this association.

## 2. Methods

### 2.1. Search Strategy

A systematic search of studies on the association of serum lipocalin-2 concentrations with psoriasis/PsA was conducted in PubMed, Web of Science, Elsevier ScienceDirect, and Cochrane Library. Keywords for the search were as follows: (“Lipocalin-2” or “LCN2”) and (“Psoriasis” or “Psoriatic arthritis” or “PsA”). The last search was updated on 27 April 2019. All relevant studies were retrieved carefully.

### 2.2. Eligibility Criteria

The inclusion criteria were (I) studies evaluated the association between serum lipocalin-2 and psoriasis/PsA; (II) case-control study; (III) studies based on human; and (IV) studies provided the detailed relevant data of both the case group and the control group. Studies were excluded if (I) the study was a review, editorial, abstract, case report, or unpublished article; (II) nonhuman studies or animal experiments or cell experiments; and (III) studies had no controls or no detailed relevant data.

### 2.3. Data Extraction

The data of the eligible studies were extracted by two investigators independently (Mr. Wang and Ms. Fang). The following information were collected: name of the first author, year of publication, country, region, ethnicity, number of cases and controls in each study, method for determining serum lipocalin-2, serum lipocalin-2 levels (Mean ± SD) in cases and controls, characteristic of controls, and other additional information. Different ethnicity descendants were classified as Caucasian and Asian.

### 2.4. Quality Assessment

The qualities of those eight eligible studies in our meta-analysis were assessed by another investigator (Dr. Pan). The quality assessment was based on the modified Newcastle-Ottawa Quality Assessment Scale (NOS). The scale consists of eight multiple-choice questions that involve subject selection, comparability in cases and controls, and assessment of exposure. High-quality response earns a point, totaling up to nine points (the comparability question earns up to two points). The higher score indicates better quality.

### 2.5. Statistical Analysis

This meta-analysis was conducted following the guidelines from PRISMA and MOOSE statement [24, 25]. All statistical analyses were performed by Stata 12.0 software (Stata Corporation, College Station, TX, USA). The strength of association between serum lipocalin-2 levels and psoriasis/PsA was assessed by the pooled standard mean difference (SMD) and its 95% confidence intervals (CIs). The *χ*^2^ test-based *Q* statistic was generally used to assess the heterogeneity. Heterogeneity was recognized as statistically significant when *I*^2^ > 50%. According to the value of *I*^2^, we chose fixed-effects model or random-effects model. All subgroups were analyzed. Sensitivity analysis was used to evaluate the influence of individual study on the overall SMD. Publication bias was assessed by Begg's test and Egger's test. The *P* value of Egger's test or Begg's test less than 0.05 was considered as significant publication bias.

## 3. Results

### 3.1. Literature Search

**Figure 1:**
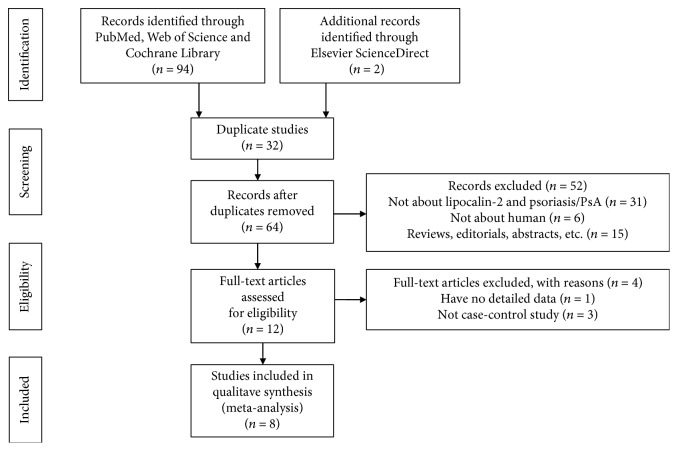
A flowchart of the included and excluded studies.

A total of 96 studies were retrieved from PubMed, Web of Science, Elsevier ScienceDirect, and Cochrane Library. Finally, 8 eligible studies were included in this meta-analysis. A flowchart of the included and excluded studies was shown in Figure 1.

### 3.2. Characteristics of the Included Studies

Table 1 shows the main features of those included studies. Those studies were published from 2012 to 2019. Eight studies involving 349 psoriasis/PsA patients and 258 controls were included in this meta-analysis. The study population was from Europe (five studies), Asia (two studies), and Middle East (one study). The serum lipocalin-2 level in one study (Baran et al.) was not shown in the form of Mean ± SD, which needed to be transformed. The original data and the data after transformation were shown in Table 2.

### 3.3. Meta-Analysis of Association between Serum Lipocalin-2 Concentrations and Psoriasis/PsA

**Figure 2:**
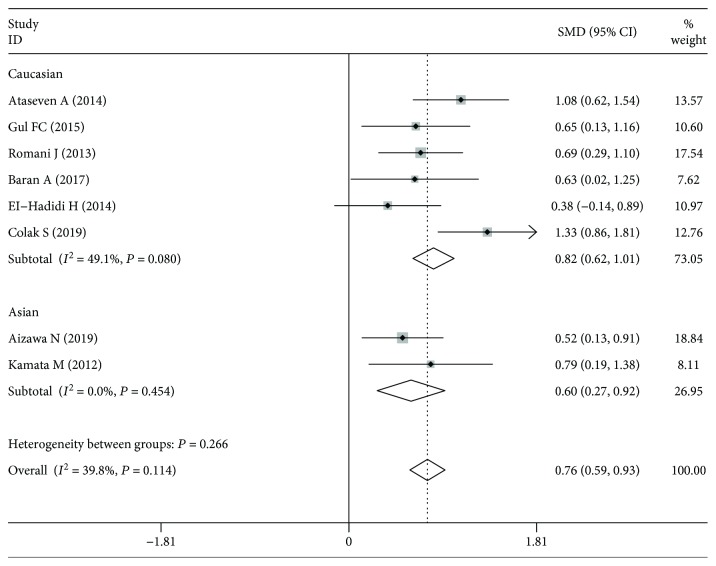
The forest plot about the association between serum lipocalin-2 levels and psoriasis/PsA.

The meta-analysis indicated the significant association between serum lipocalin-2 concentrations and psoriasis/PsA susceptibility in the overall population (SMD: 0.757, 95%CI = 0.588‐0.926, *P*_H_ = 0.114). Then, subgroup analysis was conducted according to ethnicity. The significant association was identified in Caucasians (SMD: 0.815, 95%CI = 0.618‐1.013, *P*_H_ = 0.080) and in Asians (SMD: 0.599, 95%CI = 0.273‐0.925, *P*_H_ = 0.454). The forest plot for serum lipocalin-2 concentrations with psoriasis/PsA was shown in Figure 2. The results of subgroup analysis were shown in Table 3.

### 3.4. Heterogeneity and Publication Bias

**Figure 3:**
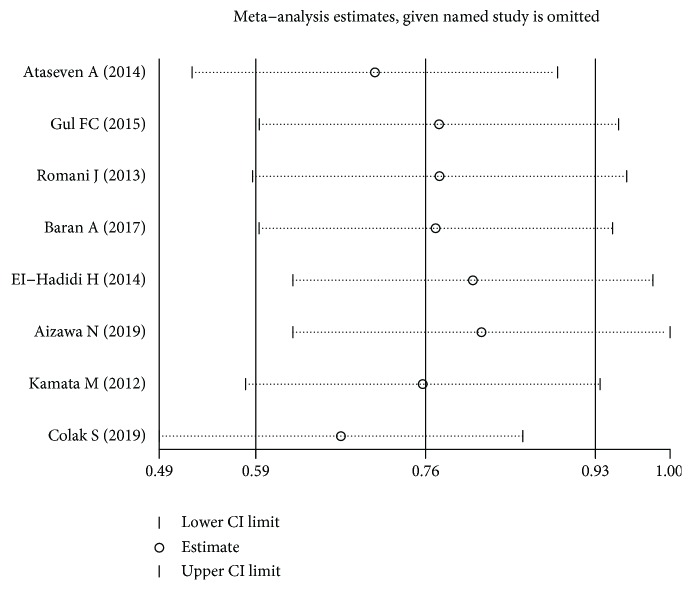
The plot of sensitivity analysis about the association between serum lipocalin-2 levels and psoriasis/PsA.

The results of heterogeneity test were shown in Table 3. Heterogeneity test showed that there was no significant difference across these studies (*I*^2^ < 50%); therefore, the fixed-effects model was performed. Publication bias was assessed by Begg's test and Egger's test. And there was no significant evidence of publication bias found in this meta-analysis (Table 3). The plot of sensitivity analysis was shown in Figure 3.

## 4. Discussion

Our meta-analysis included eight case-control studies which were about the association between serum lipocalin-2 levels and psoriasis/PsA. Our meta-analysis indicated that serum lipocalin-2 levels were significantly higher in psoriasis/PsA patients than in controls (SMD: 0.757, 95%CI = 0.588‐0.926, and *P*_H_ = 0.114; *P*_H_ is the *P* value for the heterogeneity test). Serum lipocalin-2 levels might be a potential risk factor or biomarker for psoriasis/PsA.

Lipocalin-2 (LCN2) is mainly secreted by activated neutrophils and associated with neurodegeneration, inflammatory responses, insulin resistance, and atherosclerotic disease [12]. The pathogenetic mechanisms of LCN2 in psoriasis/PsA are unknown. However, some studies have shown that LCN2 may be involved in the pathogenesis of psoriasis/PsA by modulating neutrophil function [11]. Furthermore, LCN2 may be related to neutrophil infiltration, migration and activation and epidermal differentiation [21, 22]. Psoriasis is a T-helper (Th)1/Th17-mediated, chronic inflammatory dermatosis related to metabolic syndromes. LCN2 can exacerbate psoriasiform skin inflammation by augmenting T-helper 17 response [22]. As a member of proinflammatory cytokines, it has been reported that IL-17 may be involved in the pathogenesis of psoriasis/PsA [2]. Some studies have demonstrated that IL-17 signaling pathways can induce LCN2 expression and secretion [8, 26]. Secretion of LCN2 can be regulated by IL-17 either alone or in conjunction with TNF-*α* [8, 27]. It suggests that LCN2 might play an important role in potential psoriasis/PsA pathogenesis. Some studies have shown that serum LCN2 is not related to BMI and disease activity in psoriasis/PsA patients [13, 20]. Moreover, there are conflicting results regarding the correlation between serum LCN2 and PASI [12, 15]. However, serum LCN2 level may not be a reliable indicator in the efficacy of antipsoriatic treatment [7, 23].

Of course, there were some limitations in this meta-analysis. First, the number of included studies was not sufficient to conduct comprehensive analysis. Second, our data of meta-analysis were from retrospective studies, which may be related to the methodological deficiencies. Third, our meta-analysis was primarily based on unadjusted data and did not control for confounding factors including age and gender. Therefore, it cannot exclude the influence of mixed factors. All in all, the results should be interpreted with caution.

In conclusion, the present study indicates that serum lipocalin-2 concentrations are higher in psoriasis/PsA patients than controls. However, it is still necessary to conduct more large-scale studies to further explore the pathogenetic mechanisms of LCN2 in psoriasis/PsA.

## Figures and Tables

**Table 1:** The main features of included studies.

Author	Year	Region	Country	Ethnicity	Disease	Method for determining lipocalin-2	Case			Control			Characteristic of controls	NOS score
							*n*	Mean (ng/ml)	SD	*n*	Mean (ng/ml)	SD		
Ataseven et al. [13]	2014	Europe	Turkey	Caucasian	Psoriasis	ELISA kit (BioVendor R&D Laboratorni Medicina a.s., Karasek, Czech Republic)	56	2.54	0.46	33	2.07	0.39	Healthy	7
Gul et al. [20]	2015	Europe	Turkey	Caucasian	Psoriasis	ELISA kit (BOSTER trademark, catalogue number: EK0853)	30	5.73	1.08	30	4.62	2.18	Healthy	6
Romani et al. [15]	2013	Europe	Spain	Caucasian	Psoriasis	ELISA kit (BioVendor R&D Laboratory Medicine Inc., Palackeho, Czech Republic)	50	50.34	14.42	50	40.40	14.32	NA	6
Baran et al. [7]	2017	Europe	Poland	Caucasian	Psoriasis	ELISA kit (Quantikine, R&D Systems, Minneapolis, MN, USA)	37	Median (86.00)	IQR (35.00-136.00)	15	Median (38.00)	IQR (28.00-61.00)	Healthy	6
EI-Hadidi et al. [23]	2014	Middle East	Egypt	Caucasian	Psoriasis	ELISA kit (Quantikine, R&D Systems, Minneapolis, MN, USA)	30	54.03	40.40	30	41.06	27.57	Healthy	7
Aizawa et al. [11]	2019	Asia	Japan	Asian	Psoriasis	ELISA kit (Quantikine, R&D Systems, Minneapolis, MN, USA)	59	80.08	51.30	47	59.07	20.18	Healthy	7
Kamata et al. [12]	2012	Asia	Japan	Asian	Psoriasis	ELISA kit (Quantikine, R&D Systems, Minneapolis, MN, USA)	37	41.10	14.90	17	30.70	8.10	Healthy	6
Colak et al. [8]	2019	Europe	Turkey	Caucasian	PsA	ELISA kit (double antibody sandwich ELISA method) test kit (Elabscience Biotech Co. Ltd.)	50	5.20	2.67	36	1.94	2.09	Healthy	7

**Table 2:** The characteristics of the study (Baran et al. [7]) after data transformation.

Author	Data	Mean (case)	SD (case)	Mean (control)	SD (control)
Baran et al.	Original data	Median (ng/ml)	IQR	Median (ng/ml)	IQR
		86.00	(35.00-136.00)	38.00	(28.00-61.00)
	Data after transformation	Mean (ng/ml)	SD	Mean (ng/ml)	SD
		85.64	77.90	42.72	27.00

The conversion between median, interquartile range, mean, and standard deviation is based on references [28–30].

**Table 3:** The results of meta-analysis, heterogeneity test, and publication bias.

Subgroups	*N*	SMD (95% CI)	*P*	Test of heterogeneity		Test of publication bias				Model
						Begg's test		Egger's test		
				*I* ^2^	*P*_H_	*Z*	*P*	*t*	*P*	
Overall	8	0.757 (0.588-0.926)	<0.001	39.80%	0.114	0.12	0.902	0.10	0.924	F
Ethnicity										
Caucasian	6	0.815 (0.618-1.013)	<0.001	49.10%	0.080	0.00	1.000	-0.52	0.629	F
Asian	2	0.599 (0.273-0.925)	<0.001	0.00%	0.454	0.00	1.000	NA	NA	F
Disease										
Psoriasis	7	0.673 (0.492-0.854)	<0.001	0.00%	0.530	0.00	1.000	0.13	0.903	F
Region										
Europe	5	0.893 (0.679-1.108)	<0.001	38.10%	0.167	0.24	0.806	-0.29	0.789	F
Asia	2	0.599 (0.273-0.925)	<0.001	0.00%	0.454	0.00	1.000	NA	NA	F
Original data										
Mean ± SD	7	0.767 (0.591-0.943)	<0.001	47.60%	0.075	0.30	0.764	0.29	0.782	F

## Data Availability

The data supporting this meta-analysis are from previously reported studies, which have been cited as references. The data used to support the findings of this study are included within the article. The processed data are available in Table 1 of this manuscript.
